# The magnitude of oral rehydration salt utilization in diarrhea hot spot regions of Ethiopia and its associated factors among under-five children: A multilevel analysis based on Bayesian approach

**DOI:** 10.3389/fpubh.2022.960627

**Published:** 2022-11-10

**Authors:** Yilkal Negesse, Gossa Fetene Abebe, Abebaw Addisu, Melsew Setegn Alie, Dereje Alemayehu

**Affiliations:** ^1^College of Health Science, Debre-Markos University, Debre-Markos, Ethiopia; ^2^College of Medicine and Health Science, Mizan-Tepi University, Mizan-Aman, Ethiopia

**Keywords:** Ethiopia, Ethiopian demographic health survey, children, ORS, diarrhea

## Abstract

**Background:**

Diarrhea leads the children to severe dehydration or death as a result of the loss of water and electrolytes (namely, potassium, chloride, sodium, and bicarbonate). To compensate for the losses, ORS is given to children who experienced diarrhea.

**Objective:**

To estimate the magnitude of ORS utilization in diarrhea hotspot regions of Ethiopia and to assess its associated factors among under-five children.

**Methods:**

To conduct this study, we used the 2016 Ethiopian demographic and health survey data. A total of 1,079 weighted sample children were selected. Each sample was selected randomly. Then, to identify factors associated with ORS utilization in diarrhea hotspot regions of Ethiopia, a multilevel analysis based on the Bayesian approach was applied. Finally, the credible interval of AOR that does not include 1 was considered statistically significant.

**Results:**

The magnitude of ORS utilization for children in diarrhea hotspot regions of Ethiopia was 28%. Being urban resident (AOR = 1.92; 95% CrI: 1.13–3.3), woman household head (AOR = 2.11; 95% CrI: 1.3–3.9), having higher educational level (AOR = 1.52; 95% CrI: 1.04–2.22), member of health insurance (AOR = 1.73; 95% CrI: 1.14–2.43), and being exposed for media (AOR = 1.43; 95% CrI: 1.18–2.5) increases ORS utilization for diarrhea management.

**Conclusion:**

Residence, educational level, health insurance, and media exposure were the factors of ORS utilization. So, to increase the practice of ORS utilization for diarrhea management in Ethiopia, the Ministry of Health and the Government of Ethiopia should consider those factors when they design diarrhea prevention and control strategies.

## Background

According to World Health Organization (WHO) passing loose or watery stool three or more times during 24 h is considered Diarrhea (1). An individual who experiences diarrhea has symptoms of vomiting, fever, severe watery diarrhea, and abdominal pain. This is common in children and all adults who experienced diarrhea. Those symptoms lead the children to severe dehydration or death as a result of the loss of water and electrolytes (namely, potassium, chloride, sodium, and bicarbonate). Even though the death of children over time due to diarrhea showed a significant reduction, still diarrhea remains one of the top five causes of morbidity and mortality. In Africa, it is responsible for an estimated 3,33,000 children's death and also accounts for one-fourth of all childhood deaths annually across the globe (2–6). According to different studies, lack of sanitation, poor hygiene practices, unsafe waste disposal, inadequate and unsafe water, and not being vaccinated for Rotavirus and Measles are accountable for diarrheal diseases occurrence (7–9).

The combination of electrolyte and sugar solutions was introduced in 1960 to treat water and electrolyte losses due to diarrhea (10). This combination is known as Oral Rehydration Salt (ORS). This salt is simple, affordable, and can be given at home by mothers or caregivers when the child experiences diarrhea (11, 12). Different studies showed that after the introduction of ORS, morbidity and mortality of children over time due to diarrhea have declined significantly (13–15). Even though ORS is very crucial to replace the lost fluid due to diarrhea, its utilization is still low in low- and middle-income countries (16). In 2017, the overall ORS utilization coverage in middle and low-income countries was below 50% (about 6.52 million children out of 13.34 million children with diarrhea, were not taking ORS to treat dehydration) (16).

Despite the Ethiopian ministry of health and the government collaboratively making an effort to increase the utilization of ORS for diarrhea management, only 46% of children with diarrhea received ORS (17). Also, according to the reports of the International Vaccine Access Center (IVAC), despite Ethiopia being one of the top countries in under-five children death by diarrhea, they achieved only 30% of ORS treatment targets from 90% of treatment targets in 2020 (18). According to the study conducted in Ethiopia, among the nine regional states and two city administrations, four regions (namely, Amhara, Oromia, SNNP, and Benishangul Gumuz) and one city administration (Addis Ababa) were the hotspot regions for diarrhea (19). But there are no studies conducted at the national level which show the magnitude of ORS utilization to manage diarrhea and its associated factors in diarrhea hotspot regions of Ethiopia. So, the identification of risk factors is crucial to increase the utilization of ORS for diarrhea management and to prevent and control diarrhea in diarrhea hotspot regions of Ethiopia using an appropriate statistical method of analysis. The Bayesian analysis approach is one of the data analysis approaches independent of the classical analysis approach and the parameters are estimated from the posterior distribution which is the combination of the prior information and the likelihood of the data. A prior distribution of a parameter is the probability distribution that represents our uncertainty about the parameter before the current data are examined and the likelihood function (often simply called the likelihood) expresses how probable a given set of observations is for different values of the statistical parameters. Therefore, this study aimed to estimate the magnitude of ORS utilization and to identify its associated factors among under-five children in diarrhea hotspot regions of Ethiopia by considering the clustering effect using the Bayesian analysis approach.

## Methods

### Data source and population

This study was performed based on the 2016 Ethiopian Demographic and Health Survey (EDHS) data. To access the data, we requested the Measure DHS center online by explaining the objective of our study. Then, after permission was granted, the data were accessed from the Measure DHS website (http://www.dhs_program.com). In Ethiopia, the DHS data are nationally representative and collected from 9 regions and 2 city administrations in the country every 5 years cross-sectionally. Before the data were collected, stratified two-stage sampling of clusters was carried out in each of the surveys. Each region of the country was stratified into urban and rural areas. For the 2016 survey, a total of 645 enumeration areas (EA) were selected randomly proportional to the EA size. In the second stage, on average 27–32 households per EA were selected (20–22). All under-five children in diarrhea hotspot regions of Ethiopia were the source population. In this region, all under-five children who experienced diarrhea within 2 weeks before the survey in the selected enumeration households were included in the study. Finally, a total of 1,079 weighted samples under-five children were used to conduct this study. The comprehensive procedure for sampling and sample size determination technique, in general, about the survey, is described in the complete EDHS report (20–22).

### Variables

The dependent variable is the utilization of ORS for under-five children who have diarrhea. The response was coded as “Yes = 1” and “No = 0”. The EDHS asked respondents to answer the question “Did you give ORS to your children who have diarrhea?” So, the response is binary with possible values *Y*_*i*_ = Yes if *i*^*th*^ child get ORS and *Y*_*i*_= No, if the *i*^*th*^ child had not gotten ORS.

The independent variables were classified as community- and individual-level variables. Region and place were considered as community-level factors. The number of under-five children, family size, educational level of the mother, working status of the mother, wealth status, exposure to media, sex of household head, health insurance, distance to the health facility, age and gender of the child, being twin, and the weight of the child at birth were considered as individual-level factors.

### Data processing and analysis

After the data set was downloaded from the Measure DHS website, the variables of the study were extracted from Ethiopian Demographic and Health Survey kid record (KR) data set using the software STATA version 14.2. The extracted data were weighted using sampling weight before any statistical analysis was performed to restore the representativeness of the survey and get reliable statistical estimates. Then, based on the diarrhea status (hot or cold spot) of the country, regions were categorized into a hotspot and cold spot region for diarrhea. Cold spot regions for diarrhea were excluded from the analysis. The data (data from diarrhea hotspot regions) were exported to the R software version 4.0 for further analysis. Then the data were coded, edited, and cleaned for analysis.

A multilevel model based on the Bayesian statistical approach was fitted to identify factors associated with not utilizing ORS for under-five children who have diarrhea in Ethiopia. In this study, two levels of data hierarchy were considered to fit the multilevel models. Children in the household were nested to the EA and were considered as level one units, whereas the EAs were considered as level two units.

The category of the dependent variable was dichotomous and represented as follows:


$$Y_{ij}=\{\begin{array}{c} Yes,\;if\;they\;utilized\;ORS\;for\;diarrhea\\ \;No,\;if\;they\;didn^{\prime }t\;utilized\;ORS\;for\;diarrhea\; \end{array}$$

So, this variable hass Bernoulli distribution with successes when the child utilized ORS for diarrhea and the failure when the child did not utilize ORS for diarrhea.

The probability of utilizing ORS for diarrhea for *i*^*th*^ children in the *j*^*th*^ cluster is π_*ij*_


$$P(X_{ij}=x)=\left(\begin{array}{c} n\\ x \end{array}\right)p^{x}{\left(1-p\right)}^{n-x},if\;X=\sum_{i=1}^{n}Y_{ij}\;$$

Let π_*ij*_ be modeled using a logit link function. The two-level model is given as follows:


$$Logit(\pi_{ij})=\beta_{0}+\sum_{h=1}^{h}\beta_{hj}X_{hj}+\mu_{0j}+\sum_{h=1}^{h}\mu_{hj}$$

$$\begin{array}{l} \text{Where},\beta_{0j}=\beta_{0}+\;\mu_{0j}\\ \beta_{1j}=\beta_{1}+\;\mu_{1j}\\ \beta_{hj}=\beta_{h}+\;\mu_{hj} \end{array}$$

β_0_ $+\overset{h}{\underset{h=1}{\sum }}{\beta_{hj}X}_{hj}$, is called fixed part of the model

$\mu_{0j}+\overset{h}{\underset{h=1}{\sum }}\mu_{hj}$, is called random effect of the model

*X*_*ij*_, are the covariates in the model

β, is the regression coefficient of the parameter

μ_*hj*_, is the estimate of random intercept.

We used the recently developed R package called Brms (23) to fit multilevel models. This package uses No-U-Turn Sampler (NUTS) to estimate the extent of random variations between clusters and parameters of the variable. No-U-Turn Sampler (NUTS) is an extension of HMC and it uses a recursive algorithm to build a set of likely candidate points that spans a wide swath of the target distribution, stopping automatically when it starts to double back and retrace its steps (23). When multilevel models based on the Bayesian approach are fitted using NUTS, they converge to high-dimensional target distributions much more quickly as compared with the Gibbs sampler (23).

Four models are fitted by considering flat prior with beta distribution (1, 1) for regression coefficients and gamma distribution (0.001, 0.001) for the variance, cores = 2, iteration = 8,000, burn-in iterations = 1,000, number of chains = 2, to control the divergent transition, alpha delta = 0.95, and initials = 0.

The first model (fitted without covariate) is nested into the second model (fitted with individual-level factors), the third model (fitted with community-level factors), and the fourth model (fitted with both individual- and community-level factors). Again, the second and the third models are also nested into the fourth model. A multicollinearity test was conducted and there was no multicollinearity among variables (all variables had no tolerance of < 0.1 and variance inflation factor (VIF) of >10, with a maximum VIF of 1.34). Those four models were compared based on their widely applicable information criteria (WAIC). Finally, the fourth model was selected since it has the smallest WAIC value as compared with the rest three fitted models. Therefore, we made all interpretations and inferences based on the fourth model. The intraclass correlation coefficient (ICC) value >5% was considered to determine the variation of ORS utilization for diarrhea among under-five children between the enumeration areas of diarrhea hotspot regions. Also, to declare statistically significant, we used the 95% posterior credible interval. The credible interval of AOR that included 1 was not considered statistically significant.

Finally, the convergence of the algorithms was checked using Rhat value = 1, Bulk_ESS and Tail_ESS >1,000, chains of the time series plots mixed well and smooth density plot. Because the estimates of the posterior distribution might not be reliable unless the chain of distribution has reached its stationary (24).

## Results

### Study participant characteristics

Based on the sociodemographic characteristics, 91.4% of participants were rural residents. Among the total, the majority of the study participants were from Oromia regional state (44.5%) followed by SNNP (27.9%) and Amhara regional (25%) state. Based on the birth interval, 20.2% of the children were born with narrow birth intervals (< 23 months interval). Regarding the media exposure status of the respondents, 78.3% of the participants were not exposed to the media (Table 1).

**Table 1:** Characteristic's frequency and percentage distribution of study participants in Ethiopia in 2016.

**Variables**	**Categories**	**Frequency**	**Percent**
Sex of child	Male	566	52.5
	Female	513	47.5
Twin	Yes	28	2.6
	No	1,051	97.4
Birth order	First	220	20.4
	2–3	337	31.2
	4–5	265	24.6
	≥6	257	23.8
Birth interval	≤ 23 month	174	20.2
	≥24 month	686	79.8
Child weight at birth	Normal	369	34.4
	Small	342	31.9
	Big	363	33.8
Working status of mothers	Had work	740	68.6
	Had no working	339	31.4
Wealth status	Poor	455	42.2
	Medium	250	23.1
	Rich	375	34.7
Distance to health facility	Not long	443	41.1
	Long	636	58.9
Health insurance	Yes	33	3
	No	1,047	97
Region	Amhara	270	25
	Oromia	481	44.5
	B/Gumuz	10	0.9
	SNNP	301	27.9
	Addis Ababa	18	1.6
Residence	Rural	986	91.4
	Urban	93	8.6
Sex of household head	Male	972	90
	Female	108	10
Number of U-5 children	≤ 2	953	88.3
	≥3	126	11.7
Family members	≤ 5	534	49.5
	≥6	545	50.5
Mother's educational level	No formal education	672	62.2
	Primary	335	31
	Secondary	56	5.2
	Higher	17	1.6
Media exposure	No	845	78.3
	Yes	235	21.7

### Magnitude of ORS utilization among under-five children in diarrhea hotspot regions of Ethiopia

The overall magnitude of ORS utilization for children in diarrhea hotspot regions of Ethiopia was 28%. The utilization of ORS for diarrhea management in diarrhea hotspot regions of Ethiopia is almost comparable based on different sociodemographic variables. Based on the residence, 35.5% of the rural and 27.4% of the urban residents utilize ORS for diarrhea management. Based on the regional state, Addis Ababa city administration is the highest regional state (55.6%), followed by B/Gumuz (54.5%), SNNP (33.2%), Amhara (28.5%), and Oromia (22.9%), respectively among diarrhea hotspot regions to utilize ORS for diarrhea. Regarding wealth status, 26.6%, 31.6%, and 27.5% of the households utilize ORS for diarrhea management whose wealth status is poor, medium, and rich, respectively. Similarly, based on educational level of the mothers, 26.5% of mothers who had no formal education level, 30.2% of mothers who had primary education level, 26.8% of mothers who had secondary education level, and 47.1% of mothers who had higher education level utilize ORS for diarrhea management in diarrhea hotspot regions of Ethiopia (Table 2).

**Table 2:** The magnitude of ORS utilization based on different characteristics among under five children in diarrhea hotspot regions of Ethiopia in 2016.

**Variables**	**Categories**	**Percentage**
Sex of child	Male	29
	Female	26.9
Twin	Yes	17.9
	No	28.3
Birth order	First	26.8
	2–3	29.9
	4–5	27.2
	≥6	27.6
Birth interval	≤ 23 month	20.8
	≥24 month	30.2
Child weight at birth	Normal	32.8
	Small	24.5
	Big	26.4
Working status of mothers	Had work	32.4
	Had no working	26
Wealth status	Poor	26.6
	Medium	31.6
	Rich	27.5
Distance to health facility	Not big problem	28.4
	Big problem	27.8
Health insurance	Yes	27.6
	No	39.4
Region	Amhara	28.5
	Oromia	22.9
	B/Gumuz	54.5
	SNNP	33.2
	Addis Ababa	55.6
Residence	Rural	35.5
	Urban	27.4
Sex of household head	Male	26.4
	Female	42.6
Number of U-5 children	≤ 2	28.2
	≥3	26.2
Family members	≤ 5	27.9
	≥6	28.3
Mother's educational level	No formal education	26.5
	Primary	30.2
	Secondary	26.8
	Higher	47.1
Media exposure	No	27.7
	Yes	28.9
Overall magnitude		28

### Multilevel analysis

As shown in Table 3, the variance between enumeration areas and the utilization of ORS for children who have diarrhea in diarrhea hotspot regions of Ethiopia. Therefore, the utilization of ORS across the cluster is different. In addition, the ICC in this study is 12.1%. This means roughly 12.1% of the variability of the utilization of ORS for children with diarrhea in diarrhea hotspot regions of Ethiopia was attributable to the enumeration areas. The model fitted by using community and individual variables has the smallest WAIC (3,124) among the four fitted models. Since models which have the smallest WAIC value are considered the best-fitted model (25), we made a report and interpretation based on this model.

**Table 3:** Factors associated with ORS utilization among under-five children in diarrhea hotspot regions of Ethiopia in 2016.

**Variables**	**Categories**	**AOR [95% CrI]**	**Rhat**	**Bulk_ESS**	**Tail_ESS**
β0 intercept^*^		0.14 [0.06, 0.32]	1	8,894	9,120
Sex of child	Male				
	Female	0.83 [0.61, 1.13]	1	7,867	8,567
Twin	Yes	0.64 [0.22,1.84]	1	8,934	8,645
	No				
Birth order	First				
	2–3	1.20 [0.80, 1.70]	1	9,234	9,345
	4–5	1.02 [0.68, 1.52]	1	6,784	6,657
	≥6	1.04 [0.70, 1.50]	1	7,689	7,653
Birth interval	≤ 23 month	0.60 [0.39, 0.93]	1	7,856	7,980
	≥24 month				
Child weight at birth	Normal				
	Small	0.60 [0.41, 1.03]	1	9,745	10,123
	Big	1.13 [0.71, 1.78]	1	10,231	10,545
Working status of mothers	Had work				
	Had no working	1.07 [0.76, 1.53]	1	11,234	11,543
Wealth status	Poor				
	Medium^*^	1.90 [1.40, 2.10]	1	9,764	9,985
	Rich ^*^	1.28 [1.12, 1.92]	1	8,674	8,874
Distance to health facility	Not big problem	1			
	Big problem	0.77 [0.54, 1.10]	1	6,575	6,789
Health insurance	Yes^*^	1.73 [1.14, 2.43]	1	7,123	6,997
	No				
Residence	Rural				
	Urban^*^	1.92 [1.13, 3.30]	1	8,892	9,012
Sex of household head	Male				
	Female^*^	2.11 [1.30, 3.90]	1	9,177	9,457
Number of U-5 children	≤ 2				
	≥3	1.06 [0.66, 1.71]	1	8,141	8,272
Family members	≤ 5				
	≥6	1.40 [0.91, 2.22]	1	7,459	7,359
Mother's educational level	No formal education				
	Primary^*^	1.52 [1.04, 2.22]	1	5,989	5,747
	Secondary	1.12 [0.52, 2.30]	1	6,852	6,743
	Higher^*^	3.5 [1.30, 5.00]	1	7,457	7,792
Media exposure	No				
	Yes^*^	1.43 [ 1.18, 2.50]	1	8,932	9,089
$\sigma_{\mu 0}^{2}*$		0.22 [0.05, 1.22]	1	4,896	5,231
ICC		12.1 [6.20, 18.74]			
WAIC		3,124			

* = Significant at 95% CrI.

Among the factors included in a model which contains both community- and individual-level factors, residence, wealth status, being a member of health insurance, gender of household head, educational level, and media exposure status were significantly associated with ORS utilization for children who have diarrhea in diarrhea hotspot regions of Ethiopia (Table 2). Being an urban resident, the odds of utilizing ORS is 1.92 times (AOR = 1.92; 95% CrI: 1.13–3.3) more likely as compared with rural residents. The odds of ORS utilization for children with diarrhea among middle and rich wealth status households is 1.9 and 1.28 times (AOR = 1.9; 95% CrI: 1.4–2.1), and 1.28 (AOR = 1.28; 95% CrI: 1.12–1.92) more likely as compared with poor wealth status households, respectively. Being a woman household head, the utilization of ORS for children with diarrhea is 2.11 times (AOR = 2.11; 95% CrI: 1.3–3.9). Also, the odds of the utilization of ORS for children with diarrhea among households who are exposed to the media is 1.43 times (AOR = 1.43; 95% CrI: 1.18–2.5) more likely as compared with households who are not exposed to the media.

## Discussion

Diarrhea leads an individual to many watery stools, vomiting, fever, and abdominal pain. Finally, all those manifestations may lead an individual to severe dehydration or death depending on its severity. So, to replace the lost body fluids, ORS is given to individuals who experienced diarrhea (11, 12). Of the factors included in the selected model, residence, educational level of mothers, wealth status, being a member of health insurance, sex of household head, and media exposure status were significantly associated with ORS utilization for children who have diarrhea in diarrhea hotspot regions of Ethiopia.

This study revealed that the odds of utilizing ORS among urban residents are more likely as compared with rural residents. This finding is concurrent with a study conducted in Ethiopia (26). This might be due to urban residents having a better health care service access and good knowledge about ORS in response to diarrhea as compared with Ethiopian rural residents. Since urban residents have more media exposure (such as Television, Radio, Internet, Newspaper…) than rural residents, they might have more information about ORS utilization and seek healthcare services early when their child experiences diarrhea.

The odds of utilizing ORS for children who experienced diarrhea among the mothers who have primary and higher education levels are more likely as compared with the mothers who did not have formal education. This finding is evidenced by studies conducted in Cameroon (27) and Kenya (28). This might be due to educated mothers having good knowledge about diarrhea and its complications (29–31). Therefore, educated mothers might go to health facilities early and obtain health education from healthcare professionals about the appropriate treatment of their children, including the importance, preparation, and utilization of ORS. Similarly, households whose wealth status is rich and medium utilize ORS more likely as compared with households whose wealth status is poor for their children who experienced diarrhea. This finding is concurrent to the findings of the studies conducted in India (32, 33). This is the fact that in developing countries, such as in Ethiopia, poor households cannot afford the costs of healthcare services including ORS (34). So, children of poor households might not get ORS when they experienced diarrhea. Also, the odds of utilizing ORS for children who experienced diarrhea among mothers who are member of health insurance is more likely to utilize ORS as compared with mothers who are not member of health insurance. This might be due to the fact that households who are not member of health insurance and who had no health facilities close to their area of residence may not access health care services easily. The utilization of ORS among women headed households is more likely as compared with men headed households. This finding is supported by the study conducted in Gondar town (35). This might be due to women having more health seeking behavior as compared with men (36). In addition to the above, since women are close to children care (35), they might see their child when he or she experiences diarrhea. Therefore, women headed households might use ORS more likely as compared with men headed households. The odds of the utilization of ORS for children with diarrhea among households who are exposed to media are more likely as compared with households who are not exposed to media. The finding is supported by the studies conducted in sub-Saharan countries and Bangladesh (namely, Nigeria, Burkina Faso, and Niger) (37–41). This might be due to individuals who have media exposure might get more information about the advantages of using ORS during diarrhea episode and complications of diarrhea if they did not use ORS during diarrhea episode as compared with individuals who are not exposed to media.

### Strengths and limitations of the study

Conducting a study by considering the data of all diarrhea hotspot regions of Ethiopia and fitting a multilevel model using the Bayesian approach to get fine estimates of the parameters was the strength of this study. Also, as a limitation, we cannot get some variables (for example, availability and accessibility of ORS, knowledge and attitude of the participants toward ORS) to identify risk factors of ORS utilization.

## Conclusion and recommendations

The magnitude of ORS utilization for children in diarrhea hotspot regions of Ethiopia is very low. Not utilizing ORS for diarrhea management had been a major public health problem if the problem continues in this way in the study area. Therefore, all individuals whose children are experiencing diarrhea should give priority attention to utilize ORS. Being an urban resident, wealthy, educated, exposed to media, a member of health insurance, and women headed household increases the utilization of ORS for children who have diarrhea in diarrhea hotspot regions of Ethiopia. Therefore, to increase the utilization of ORS for diarrhea management in diarrhea hotspot regions of Ethiopia, all the concerned bodies including the Ethiopian Ministry of Health and Government should consider those factors when they design diarrhea prevention and control strategies specifically in diarrhea hotspot regions of Ethiopia.

## Data availability statement

The raw data supporting the conclusions of this article will be made available by the authors, without undue reservation.

## Ethics statement

Ethical clearance for the EDHS data was given by the Ethiopian Health and Nutrition Research Institute (EHNRI) Review Board; the National Research Ethics Review Committee (NRERC) at the Ministry of Science and Technology (34). To collect the data, the respondents were informed about the survey and a verbal consent was taken for their participation (34). Before the data collection, all the procedures were reviewed and approved by the ICF Institutional Review Board (34).

## Author contributions

YN, GFA, AA, MSA, and DA were participated in this study from the initial till the end, participated in design, acquisition of data, data cleaning, coding, data analysis, interpretation, drafting, and finally revising of the manuscript. All authors contributed to the article and approved the submitted version.

## Conflict of interest

The authors declare that the research was conducted in the absence of any commercial or financial relationships that could be construed as a potential conflict of interest.

## Publisher's note

All claims expressed in this article are solely those of the authors and do not necessarily represent those of their affiliated organizations, or those of the publisher, the editors and the reviewers. Any product that may be evaluated in this article, or claim that may be made by its manufacturer, is not guaranteed or endorsed by the publisher.

## Abbreviations

AOR: Adjusted Odds Ratio
CrI: Credible interval
DHS: Demographic Health Survey
EA: Enumeration Area
EDHS: Ethiopian Demographic and Health Survey
ICC: Intraclass Correlation Coefficient
ORS: Oral Rehydration Salt
SNNP: South Nation Nationality and People
WAIC: Widely Applicable Information Criteria
WHO: World Health Organization.
